# Retraction: Antitumor Activity of Sorafenib in Human Cancer Cell Lines with Acquired Resistance to EGFR and VEGFR Tyrosine Kinase Inhibitors

**DOI:** 10.1371/journal.pone.0298013

**Published:** 2024-01-25

**Authors:** The PLOS ONE Editors

Following the publication of this article [1] and the subsequent Expression of Concern [2] further concerns were raised regarding Figures 3 and 4. Specifically:

- In Figure 3 the following panels appear similar to panels found in [3]:
  - The HCT-116 CRAF western blot panel appears similar to the Pemetrexed P27 panel in Figure 3 of [3].
  - The HCT-116 p338-CRAF western blot panel appears similar to the left side BCL-2 panel in Figure 4A of [3].
  - The CALU-3 beta-actin western blot panel appears similar to the right side beta-actin panel in Figure 4A of [3].
- In Figure 4B the top left region of the CALU-3 ERL-R panel appears similar to the central region of CALU-3 VAN-R panel despite representing different experimental conditions.

The authors stated that the original underlying data for these figures was no longer available. As such the above concerns cannot be resolved.

In light of the concerns affecting multiple figure panels that question the integrity and reliability of these data, the *PLOS ONE* Editors retract this article.

FM did not agree with the retraction. EM, TT, MO FDV and FC either did not respond directly or could not be reached.
